# *In vivo* prostate IMRT dosimetry with MOSFET detectors using brass buildup caps

**DOI:** 10.1120/jacmp.v7i4.2278

**Published:** 2006-11-28

**Authors:** Raj Varadhan, John Miller, Brenden Garrity, Michael Weber

**Affiliations:** ^1^ Minneapolis Radiation Oncology 550 Osborne Road Fridley Minnesota 55432; ^2^ Radiation Therapy Department Methodist Hospital 6500 Excelsior Blvd. St. Louis Park Minnesota 55426 U.S.A.

**Keywords:** MOSFET, *in vivo* dosimetry, brass buildup caps, quality assurance, IMRT

## Abstract

The feasibility of using dual bias metal oxide semiconductor field effect transistor (MOSFET) detectors with the new hemispherical brass buildup cap for *in vivo* dose measurements in prostate intensity‐modulated radiotherapy (IMRT) treatments was investigated and achieved. In this work, MOSFET detectors with brass buildup caps placed on the patient's skin surface on the central axis of the individual IMRT beams are used to determine the maximum entrance dose $\left(D_{\max}\right)$ from the prostate IMRT fields. A general formalism with various correction factors taken into account to predict $D_{\max}$ entrance dose for the IMRT fields with MOSFETs was developed and compared against predicted dose from the treatment‐planning system (TPS). We achieved an overall accuracy of better than ±5% on all measured fields for both 6‐MV and 10‐MV beams when compared to predicted doses from the Philips Pinnacle^3^ and CMS XiO TPSs, respectively. We also estimate the total uncertainty in estimation of MOSFET dose in the high‐sensitivity mode for IMRT therapy to be 4.6%.

PACS numbers: 87.53Xd, 87.56Fc

## I. INTRODUCTION

Routine intensity‐modulated radiotherapy (IMRT) quality assurance in most institutions only involves verifying the optimized fluence map delivered to the patient in a test phantom at a certain preset depth. Reconstruction of actual patient dose delivered to the patient through electronic portal imaging systems is still in its infancy. In this scenario, verification of actual dose delivered to the patient through *in vivo* dosimetry can play a vital part in the entire chain of the quality assurance process. Metal oxide semiconductor field effect transistor (MOSFET) dosimeters with plastic water buildup caps have been in use in our institution for some time to verify routine patient entrance $D_{\max}$ doses, and we have had excellent agreement with predicted doses as reported by other groups.^(^ ^1^ ^–^ ^3^ ^)^ More recently, Thomson Nielsen, the manufacturer of MOSFETs, has introduced single, wide‐energy hemispherical buildup caps for patient dosimetry that have full buildup at $D_{\max}$ for all photon energies.

We have developed a general formalism with all the correction factors taken into account to predict $D_{\max}$ entrance dose for anterior IMRT fields

## II. MATERIALS AND METHODS

**Figure 1:**
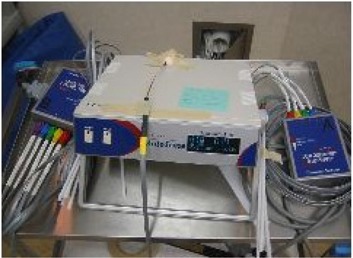
MOSFET AutoSense System

We used the MOSFET AutoSense dose verification system, model TN‐RD‐60‐W10, from Thomson Nielsen (Ottawa, Canada) for this study. The system consists of dual bias MOSFETs, model TN‐502‐RD, and a dual bias supply box, model TN‐RD‐22 (Fig. 1). One must be connected to a PC to output the data from the AutoSense reader via the AutoSense PC software program, TN‐RD‐49, which allows the user to generate a report for the patient's record. The dual bias supply system allows the user to select different modes of sensitivity (standard or high). With standard sensitivity, conversion factors are around 1 cGy/mV, and for high sensitivity conversion factors are around 3 cGy/mV. The system is powered by a 9‐V battery (two batteries are required if using the high‐sensitivity mode).

The physical characteristics of MOSFETs and the principles of dual bias detector operation are described in great detail elsewhere.^(^ ^1^ ^,^ ^3^ ^,^ ^4^ ^)^ In our clinical practice, we currently use a dual P‐channel enhancement mode type MOSFET.

The dual MOSFET used in this study consists of two MOSFETs on a silicon layer with different bias voltages applied to the gate. The dose measured in this case is then the ratio of the voltage shifts for the two MOSFETs. This is an advantage over single MOSFETs, which suffer from temperature and dose rate dependence. These effects are normalized in the dual MOSFET, and they essentially show no temperature effects as reported elsewhere.^(^ ^3^ ^,^ ^5^ ^)^

**Figure 2:**
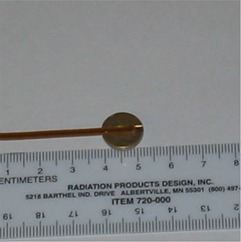
The brass buildup cap has a radius of 0.635 cm and a special groove insert for MOSFET placement, which improves accuracy of placement.

We used the wide‐energy hemispherical brass buildup caps for this study. A single brass buildup cap was used for both 6‐MV and 10‐MV beams. Due to its high density $\left(8.5\;g/{\text{cm}}^{3}\right)$ and high atomic number, brass provides the minimal amount of metal needed to achieve full buildup at $D_{\max}$ for a range of photon energies. The buildup caps are small, with a radius of only 0.635 cm. They have a special groove insert for MOSFET placement, which, we believe, improves placement accuracy and saves on patient setup time (Fig. 2). The brass buildup caps were carefully aligned to the central axis of the individual IMRT beams on the patient's skin surface before *in vivo* measurements were taken.

Our prostate IMRT delivery method involves a step‐and‐shoot technique using a Varian 21 EX linear accelerator and seven equally spaced beams starting with the AP beam. *In vivo* dosimetry was performed on five (anterior fields) of the seven fields. We achieved an overall accuracy greater than or equal to ±5% on all measured fields. We report the data here for the first 11 patients in our institution.

The general equation to calculate expected dose for *in vivo* IMRT measurement is given by expected MOSFET dose (cGy) =

$$\begin{array}{l} \text{expected}\;\text{MOSFET}\;\text{dose}\;(\text{cGy})=\\ \;D_{\text{max}}\text{TPS}\;\text{dose}\;(\text{cGy})\times \text{CR}\;\text{value}\times {\text{CF}}_{\text{SSD}}\times {\text{CF}}_{\text{IMRT}}\times \text{ISF}\;--1, \end{array}$$

where ISF is the inverse square factor, which corrects for the changes in the source‐to‐skin distance (SSD) from the planned IMRT treatment plan to the actual SSD on the day MOSFET was placed. The other correction factors are described in detail below.

We now present a general formalism to predict for $D_{\max}$ entrance skin dose from prostate IMRT beams with various correction factors .

### A. Determination of conversion factor.

Each new set of MOSFETs must be calibrated to determine the device's conversion factor (CF) value. This provides the user with the millivolt (mV) to centigray (cGy) conversion. Typical values are approximately 1 mV/cGy for standard sensitivity and 3 mV/cGy for high sensitivity. All our measurements were performed under high sensitivity.

We performed our calibration under full‐scatter conditions for the 10 MV beam; that is, the MOSFETs are placed at a $D_{\max}$ depth under full buildup (bolus and solid water measuring $30\times 30\;{\text{cm}}^{2}$) at 100 cm SSD and field size of $10\times 10\;{\text{cm}}^{2}$.

Five measurements were made with each MOSFET after delivering 50 cGy (adjusted for LINAC output just prior to calibration). The standard deviation of the detector ranged from 0.3% to 1.3%. The AutoSense system allows the user to store these conversion factors in the software program. It should also be noted that the response of MOSFETs changes over time as reported by Chuang et al.,^(4)^ who showed that the uncertainty in the readout was ±3% over a two‐week period.

An alternative method of generating CF values is to perform the calibration with the buildup caps. This is more time‐consuming because each MOSFET has to be calibrated individually. A CF value measured this way is specific to the beam energy used for the calibration as well as the type of cap used. The advantage of this method is that the additional correction factor (CR) described below is built into the CF value. Our IMRT measurements using 6‐MV beams were calibrated this way (CR=1.0), while the measurements with the 10‐MV beams were calibrated under a full‐scatter environment without the brass buildup caps.

### B. Determination of the CR value

CR values correct for the scatter environment changes when buildup caps are used when the original calibration was performed under full‐scatter conditions without the brass buildup caps. Mathematically, the CR value is the dose measured by the MOSFET under buildup cap at 100 cm SSD divided by the dose measured by the chamber at $100\;\text{cm}+D_{\max}$ by delivering the same known dose to both under standard reference conditions:

$$\text{CR}=\frac{\text{dose}\;(\text{MOSFET}(\text{buildup}\;\text{cap})}{\text{dose}\;(\text{ion}\;\text{chamber})}$$

A CR value measured this way incorporates small corrections needed in the inverse square effect (SSD to $\text{SSD}+D_{\max}$), and the changes in the scatter environment due to the electron density change with the presence of the brass buildup cap. The reference dose with chamber was measured using the Exradin A1 miniature shonka thimbe chamber connected to a CNMC model 206 electrometer.

Typical measured values are reported in Table 1.

**Table 1:** Correction factors (CR) for MOSFETs with different buildup caps

Cap	Energy	CR value
plastic water (r=1.5 cm)	6 MV	1.000
plastic water (r=2.0 cm)	10 MV	1.008
brass	10 MV	1.038
brass	6 MV	0.92

It should be noted that for our clinical patient data CR value=1.0 for the 6‐MV beam since the original calibration was done with brass buildup caps as discussed in Section A.

### C. Energy dependence

We investigated the energy dependence of MOSFET detectors for both photon energies used in our institution: 6 MV and 10 MV. A set of four MOSFETs was calibrated with both 6‐MV and 10‐MV beams on the same day after measuring the machine output. The MOSFETs were placed under $D_{\max}$ depth under full buildup conditions at 100 cm SSD; a field size of $10\times 10\;{\text{cm}}^{2}$ was used for the measurements. Three readings were taken for both energies. After analysis of data we found a difference of less than 1% in the CF for both energies. So a single CF value was used for both energies.

### D. Dose reproducibility

**Figure 3:**
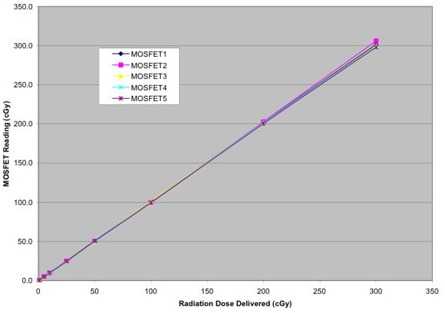
MOSFET linearity response for doses ranging from 1 cGy to 300 cGy

**Figure 4:**
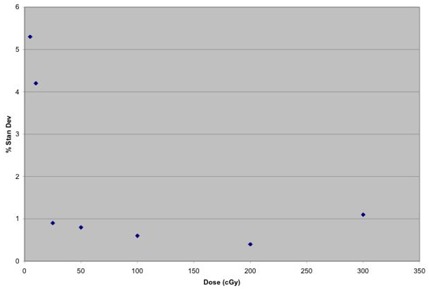
Dose delivered versus percent standard deviation

We investigated the dose reproducibility of the MOSFETs to examine the linearity of MOSFET response with dose and, in particular, the minimum dose that can be measured in relation to the sensitivity of the MOSFETs. Known doses were delivered to five MOSFETs ranging from 1 cGy to 300 cGy. The results of five dosimeter readings were averaged, and then the percent standard deviation was computed for each known dose. The results are summarized in Figs. 3 and 4.

The MOSFET response exhibits good linearity from 1 cGy to 300 cGy. The results also indicate that the minimum dose that can be measured in relation to the sensitivity of MOSFETs is around 25 cGy, where the percent standard deviation falls below 1%, which is consistent with the results reported elsewhere.^(4)^ At a low dose of 5 cGy, our percent standard deviation was 5.3% compared to a standard deviation of 20% for the same dose, as reported in the literature.^(4)^ This is because our measurements were made in the high‐sensitivity mode, while the data in Ref. 4 were collected in the low‐sensitivity mode. Based on these measurements, it can be concluded that the correction factors measured by delivering doses greater than 25 cGy are reliable.

### E. Dose rate dependence

We investigated whether the responses of MOSFET detectors are dose rate independent by taking readings at three different SSDs (120 cm, 100 cm, and 80 cm) at two different dose rates (80 MU/min and 400 MU/min). Fifty centigrays was delivered each time with a field size of $10\times 10\;{\text{cm}}^{2}$.

Our results indicated a difference of less than 1% for the two different dose rates; hence, the responses of MOSFETS are essentially dose rate independent.

### F. SSD correction factors (CFSSD)

Two MOSFETs were used at each SSD, each taking three readings. We measured data over the range of 75 cm to 120 cm SSD with the MOSFETs covered with the brass buildup cap. From 75 cm to 100 cm SSD, we measured in 5‐cm increments. Three measurements with an ion chamber were also taken at each SSD. Fifty centigrays was delivered for all measurements. All readings were performed in a $10\times 10\;{\text{cm}}^{2}$ field size. The MOSFET readings were averaged, and then we took the ratio of MOSFET to chamber dose and normalized the data to 100 cm SSD.

**Figure 5:**
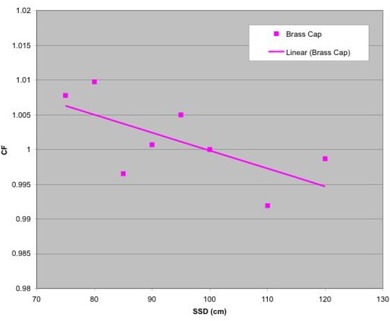
Correction factors plotted versus SSD

As illustrated in Fig. 5, the SSD correction factors are quite small for the range of clinical SSDs from 75 cm to 120 cm.

### G. IMRT patient‐specific correction factor

The final step in deriving the expected dose for *in vivo* IMRT measurements involves the computation of the IMRT patient‐specific correction factor $\left({\text{CF}}_{\text{IMRT}}\right)$ for each individual IMRT field. The ${\text{CF}}_{\text{IMRT}}$ was derived by transferring the patient treatment plan to the solid water phantom by the treatment‐planning system (TPS), then the measurements were made on the phantom. The ${\text{CF}}_{\text{IMRT}}$ factor is defined as the ratio of dose measured by MOSFET with the brass buildup cap to the dose measured by the chamber at $D_{\max}$. These measurements were all performed at a nominal gantry angle of 180± (AP) for each of the IMRT beams.

$${\text{CF}}_{\text{IMRT}}=\frac{\text{dose}\;\text{measured}\;\text{by}\;\text{MOSFET}}{\text{dose}\;\text{at}\;D_{\text{max}}\;\text{measured}\;\text{by}\;\text{chamber}}\times \frac{1}{\text{CR}\;\text{value}}\;\text{for}\;\text{each}\;\text{field}$$

It should be noted that since both the CR value and the ${\text{CF}}_{\text{IMRT}}$ represent the ratio of dose measured with MOSFET to the dose measured with ion chamber, and since the CR value represents the correction factor more suitable for standard conditions (for full buildup calibration as in the 10‐MV beam), the CR value is factored out in the computation of IMRT patient‐specific correction factor to ensure that this factor is not counted twice. For 6‐MV beams, since the original calibration was done with buildup caps, the CR value is 1.0, as discussed before.

The IMRT correction factor represents a perturbation in MOSFET measurements at $D_{\max}$ for small field sizes and leaf sequences encountered in IMRT delivery when compared to the predicted dose from the TPSs. We also verified the dose predicted at $D_{\max}$ by our TPS (CMS XiO, v4.2.2) for the first four patients, where the individual field dose varied from 26.3 cGy to 78.5 cGy with independent chamber measurements with the Exradin A1 miniature shonka thimble chamber, which has a collecting volume of 0.056 cm^3^. Our measurements agreed with the TPS calculated dose within 2% or 1 cGy. Consequently, for all future *in vivo* measurements, dose from the TPS was used as a reference, and the IMRT patient specific factor is defined as

$${\text{CF}}_{\text{IMRT}}=\frac{\text{dose}\;\text{measured}\;\text{by}\;\text{MOSFET}}{\text{dose}\;\text{at}\;D_{\text{max}}\;\text{from}\;\text{TPS}}\times \frac{1}{\text{CR}\;\text{value}}\;\text{for}\;\text{each}\;\text{field}$$

This was done mainly to avoid the extremely time‐consuming computation of ${\text{CF}}_{\text{IMRT}}$, since it would involve delivering the patient plan independently twice to calculate the MOSFET dose and the chamber dose. The user must verify that the dose predicted at $D_{\max}$ by the TPS agrees reasonably well with chamber measurements before using dose at $D_{\max}$ from the TPS as a reference in the computation of the patient‐specific IMRT correction factor.

## III. RESULTS AND DISCUSSION

We present the data in two tables. Table 2 shows *in vivo* dosimetry data using 10‐MV photons, which used full buildup calibration and the CMS XiO TPS, and data in Table 3 refer to *in vivo* dosimetry using 6‐MV photons and the Philips Pinnacle^3^ TPS. All IMRT treatments were delivered using the Varian 21 Ex linear accelerator in the step‐and‐shoot mode.

**Table 2:** In vivo measurements using MOSFETs compared to corrected target doses from the TPS (CMS XiO). All plans delivered with beam energy 10 MV. (CR=1.038)

Patient	Field #	$D_{\max}$ Dose TPS (cGy)	IMRT CF	Total CF	Expected MOSFET dose (cGy)	Measured MOSFET dose (cGy)	% Diff
	2	74.6	0.996	1.036	77.3	78.5	1.6%
#1	3	49.0	0.992	1.049	51.4	52.6	2.2%
	4	26.7	0.975	1.020	27.3	28.7	5.1%
	5	43.7	0.977	1.024	44.7	46.1	3.1%
	6	84.2	1.012	1.053	88.6	91.1	2.8%
**Total**		**278.2**			**289.3**	**296.9**	**2.6%**
	2	61.1	1.018	1.071	65.4	65.2	−0.4%
#2	3	44.7	0.996	1.009	45.1	44.0	−2.4%
	4	34.3	1.026	1.044	35.8	35.8	−0.1%
	5	39.1	1.004	1.017	39.8	39.3	−1.3%
	6	62.8	0.991	1.069	67.1	70.0	4.3%
**Total**		**242.0**			**253.3**	**254.3**	**0.4%**
	2	74.6	0.996	1.023	76.3	77.8	1.9%
#3	3	49.0	0.992	1.044	51.2	48.8	−4.7%
	4	26.7	0.975	1.027	27.5	26.3	−4.2%
	5	43.7	0.977	1.044	45.6	45.7	0.3%
	6	84.2	1.012	1.072	90.2	87.4	−3.1%
**Total**		**278.2**			**290.7**	**286.0**	−1.6%
	2	62.9	1.001	1.039	65.3	65.1	−0.4%
#4	3	49.6	0.993	1.036	51.4	51.0	−0.7%
	4	32.0	0.953	0.998	31.9	33.2	4.0%
	5	40.3	0.987	1.022	41.2	41.6	1.0%
	6	62.9	1.031	1.101	69.2	65.8	−5.0%
**Total**		**247.7**			**259.1**	**256.7**	−0.9%
	2	92.9	0.928	0.982	91.2	90.2	−1.1%
#5	3	51.4	0.965	0.985	50.7	52.4	3.3%
	4	31.5	0.989	1.014	31.9	30.4	−4.7%
	5	50.5	1.003	1.023	51.6	51.0	−1.2%
	6	92.4	0.942	1.020	94.2	103.7	10.1%
**Total**		**318.7**			**319.6**	**327.7**	**2.5%**
	2	73.1	0.973	1.010	73.8	75.0	1.6%
#6	3	52.7	0.976	0.996	52.6	51.4	−2.3%
	4	22.5	0.928	0.954	21.4	22.6	5.6%
	5	45.7	0.964	0.984	45.0	47.0	4.4%
	6	78.3	0.980	1.036	81.1	85.1	5.0%
**Total**		**272.3**			**273.9**	**281.1**	**2.6%**

**Table 3:** In vivo measurements using MOSFETs compared to corrected target doses from the TPS (Philips Pinnacle^3^). All plans delivered with beam energy 6 MV. (CR=1.0)

Patient	Field #	$D_{\max}$ Dose TPS (cGy)	IMRT CF	Total CF	Expected MOSFET dose (cGy)	Measured MOSFET dose (cGy)	% Diff
	2	82.5	0.971	0.984	81.2	87.4	7.6%
#7	3	53.2	1.006	1.027	54.6	54.0	1.2%
	4	41.8	0.995	1.018	42.6	42.6	0.1%
	5	54.2	0.984	1.005	54.5	56.3	3.4%
	6	84.0	0.976	0.988	83.1	87.4	5.2%
**Total**		**315.7**			**316.0**	**327.7**	**3.7%**
	2	105.0	0.983	0.989	103.9	109.0	4.9%
#8	3	49.9	0.973	1.035	51.6	53.2	3.0%
	4	36.0	0.979	1.022	36.8	35.4	3.8%
	5	48.5	0.971	1.032	50.1	52.6	5.1%
	6	91.6	0.972	0.985	90.2	93.5	3.6%
**Total**		**331.0**			**332.6**	**343.7**	**3.3%**
	2	112.0	0.976	0.976	109.4	115.0	5.2%
#9	3	46.3	0.951	1.006	46.6	48.2	3.5%
	4	21.9	0.944	0.983	21.5	22.7	5.4%
	5	59.3	0.965	1.009	59.8	63.0	5.3%
	6	118.3	0.967	1.073	126.9	121.0	4.7%
**Total**		**357.8**			**364.2**	**369.9**	**1.6%**
#10	2	40.2	0.981	1.022	41.1	39.3	4.4%
prosthesis	3	54.0	0.932	0.960	51.8	52.2	0.7%
(#6 not	4	66.8	0.966	0.977	65.3	65.7	0.6%
measured)	5	89.2	0.951	0.975	87.0	88.0	1.2%
**Total**		**250.2**			**245.2**	**245.2**	**0.0%**
	2	81.5	0.958	0.961	78.3	80.4	2.6%
#11	3	72.8	0.971	0.991	72.1	68.3	5.3%
	4	43.5	0.957	0.979	42.6	41.5	2.6%
	5	70.1	0.984	1.032	72.4	68.8	4.9%
	6	82.4	0.982	1.009	83.2	86.4	3.9%
**Total**		**350.3**			**348.6**	**345.4**	**0.9%**

From Tables 2and 3, the maximum overall deviation of measured MOSFET reading from the predicted dose was only 3.7% as seen in patient #7, and the maximum deviation in any one particular IMRT field (out of 54 total IMRT fields) was 10.1%, for patient #5. The main sources of discrepancy observed in our data between the TPS calculated dose and MOSFET readings could be due to uncertainty in the MOSFET response over a long period of time (±3%), errors in positioning of the brass buildup cap, patient movement, and the fact that the patient‐specific IMRT correction factor was derived with a nominal gantry angle of 180±, which is different from the actual treatment gantry angle.

Our clinical data are still well within the overall uncertainty in the calculation of MOSFET dose for IMRT therapy. We estimate the total uncertainty in the estimation of the MOSFET dose in the high‐sensitivity mode for IMRT therapy to be 4.6 %, as described in Table 4.

**Table 4:** Uncertainty of the MOSFET dose for IMRT therapy given as 1 SD (high sensitivity)

	Uncertainty (%)
**Step 1: MOSFET calibration (Ref Cond)**	
Dose measured with ionization chamber	1.0%
Reading of the MOSFET detector	1.3%
Reproducibility of the setting	0.5%
Combined uncertainty of step 1	1.7%
**Step 2: Correction factors**	
SSD correction factor	1.4%
CR value	1.5%
IMRT correction factor–MOSFET reading on solid water phantom	1.3%
Combined uncertainty of step 2	2.4%
**Step 3: Measurement on the patient**	
Reading of the MOSFET detector	1.3%
Positioning (2% over 2 mm radius)	1.5%
MOSFET detector long‐term stability over time	3.0%
Combined uncertainty of step 3	3.6%
**Combined uncertainty of steps** 1+2+3	**4.6%**

Our standard deviations in the measurement of the SSD correction factor and the CR value based on raw data measurements are different than those reported by Jornet et al.^(5)^

## IV. CONCLUSION

The feasibility of using new brass buildup caps for *in vivo* prostate IMRT dosimetry was investigated and achieved. The new brass buildup caps, because of their small size and groove design, improve accuracy in positioning and take less patient setup time. We have derived a general formalism with a patient‐specific IMRT correction factor $\left({\text{CF}}_{\text{IMRT}}\right)$ to predict expected $D_{\max}$ entrance dose using these brass buildup caps for anterior IMRT fields. We have achieved an overall accuracy of better than ±5% on all measured fields for both 6‐MV and 10‐MV beams when compared to predicted doses from the Philips Pinnacle^3^ and CMS XiO treatment planning systems, respectively. We have estimated the total uncertainty in estimation of MOSFET dose in the high‐sensitivity mode for IMRT therapy to be 4.6%.

We believe that integration of *in vivo* dosimetry along with regular fluence map verification in phantoms at a preset depth using films or diode array and routine multileaf collimator quality assurance offers a greater accuracy and confidence in actual dose delivered to the patient.

## ACKNOWLEDGMENTS

One of us (JM) would like to thank Dr. John Broadhurst, Dr. Pat Higgins, Dr. Russ Ritenour, and Dr. Bruce Gerbi, faculty members at University of Minnesota, for their help and support.
